# Investigation of per- and polyfluoroalkyl substances (PFAS) in soils and sewage sludges by fluorine K-edge XANES spectroscopy and combustion ion chromatography

**DOI:** 10.1007/s11356-021-17838-z

**Published:** 2021-12-03

**Authors:** Philipp Roesch, Christian Vogel, Thomas Huthwelker, Philipp Wittwer, Franz-Georg Simon

**Affiliations:** 1 Bundesanstalt für Materialforschung und -prüfung (BAM), Division 4.3 Contaminant Transfer and Environmental Technologies, Unter den Eichen 87, 12205 Berlin, Germany; 2 Paul Scherrer Institute, Swiss Light Source, 5232 Villigen PSI, Switzerland

**Keywords:** X-ray absorption near-edge structure (XANES) spectroscopy, Extractable organically bound fluorine (EOF), Total fluorine (TF), Sum parameter analysis, Persistent organic pollutants (POPs)

## Abstract

For the first time, fluorine K-edge X-ray absorption near-edge structure (XANES) spectroscopy was applied to detect per- and polyfluoroalkyl substances (PFAS) in various soil and sewage sludge samples. The method can be used to determine the speciation of inorganic and organic fluorides, without pre-treatment of solid samples. Therefore, XANES spectra of several inorganic fluorides as well as selected fluorinated organic compounds were recorded. While inorganic fluorides partially exhibit a variety of sharp spectral features in the XANES spectrum, almost all inspected organofluorine compounds show two distinct broad features at 688.5 and 692.0 eV. Moreover, the peak intensity ratio 688.5 eV/692.0 eV in the PFAS XANES spectrum can be inversely correlated to the chain length of the perfluoro sulfonic acid group. The detection of targeted PFAS by bulk-XANES spectroscopy in combination with linear combination fitting in soils and sewage sludges was not applicable due to the low organic fluorine to total fluorine ratio of the samples (0.01–1.84%). Nonetheless, direct analysis of pure PFAS revealed that analysis of organofluorine species might be achieved in higher concentrated samples. Furthermore, quantitative measurements by combustion ion chromatography (CIC) evaluated as sum parameters extractable organically bound fluorine (EOF) and total fluorine (TF) emphasize that besides soils, sewage sludges are a significant source of organic fluorine in agriculture (154–7209 µg/kg).

**Supplementary Information:**

The online version contains supplementary material available at 10.1007/s11356-021-17838-z.

## Introduction

Per- and polyfluoroalkyl substances (PFAS) have emerged over the course of the last 20 years as a global pollution issue (Giesy and Kannan 2001; Lindstrom et al. 2011; De Silva et al. 2021). Originally designed and produced as substantially effective lipophobic and hydrophobic surface coatings and additives for industrial purposes (Kissa 2001), PFAS quickly found use in numerous consumer applications like cosmetics (Schultes et al. 2018), fast-food packaging (Schaider et al. 2017; Schultes et al. 2019), coatings on cooking ware or outdoor clothing (Hill et al. 2017; Schellenberger et al. 2019; Glüge et al. 2020). Over the last decades, increasing production, consumption, and disposal has led to wider distribution of PFAS in the environment (Stoiber et al. 2020; Jacob et al. 2021), and numerous cases of contaminated sites were reported worldwide (Sunderland et al. 2019; Kotthoff et al. 2020). A major environmental impact can be traced back to the ubiquitous use of PFAS mixtures as highly effective aqueous film forming foams (AFFFs) (Leeson et al. 2021), predominantly on firefighting training grounds (Kärrman et al. 2011; Baduel et al. 2015; Mumtaz et al. 2019; Nickerson et al. 2020). Altogether, there are currently more than 4700 known and partly characterized fluorinated compounds identified by the Organisation for Economic Co-operation and Development (OECD), most of them classified as toxic or dangerous for the environment (OECD 2018).

Due to their highly stable C–F bond (*D*_0_ = 485 kJ/mol), most PFAS exhibit a chemically inert character, making them difficult for microorganisms to degrade and inaccessible for classical environmental degradation processes like oxidation (Liu and Mejia Avendano 2013; Roesch et al. 2020). The persistence and toxicity of especially C_8_–C_14_ per- and polyfluorinated carboxylic and sulfonic acids and their respective salts lead to their inclusion to the list of regulated substances within the EU (Lallas 2017). Since addition to the group of banned persistent organic pollutants (POP) at the Stockholm Convention in 2009 and 2020, perfluorooctanesulfonic acid (PFOS), perfluorooctanoic acid (PFOA), and other long-chained molecules are often referred to as “legacy PFAS” (Sun et al. 2016). As a consequence, manufacturers moved to the production of short-chain (C_4_–C_7_) and ultrashort-chain (C_1_–C_3_) PFAS (Ateia et al. 2019) and differently fluorinated derivatives like GenX (C_3_F_7_OCF(CF_3_)COONH_4_) and ADONA (C_7_H_5_F_12_NO_4_) for commercial production and application (Lindstrom et al. 2011). In many cases, PFAS substitutes of shorter chain length exhibit similar chemical and physical properties as their longer homologues, but even less is known regarding their biocompatibility, toxicity, and persistence. The ongoing production of new, yet unrestricted, PFAS alternatives has become a major challenge not only for researchers across the planet, but also for environmental routine analytics, since the state-of-the-art method LC–MS/MS relies on structural information and availability of isotope standards of the targeted compound (Nakayama et al. 2019). Although establishment of the total oxidizable precursor (TOP) assay protocol significantly improves their analytical range (Houtz and Sedlak 2012; Janda et al. 2019), target analytical methods only enable detection of approximately 70 different PFAS, thus will increasingly become a limiting factor (Koch et al. 2020). Since the number of PFAS alternatives is also relative to advancing progress of the regulatory enforcement, different future analytical approaches are inevitable. First reported by Miyake *et al.* in 2007 (Miyake et al. 2007), fluorine sum parameters like adsorbable organic fluorine (AOF), extractable organic fluorine (EOF), and total fluorine (TF) can be applied to survey and detect the presence of large amounts of unidentified organofluorine compounds in environmental matrices like water sources (Wagner et al. 2013; Willach et al. 2016; von Abercron et al. 2019; Gehrenkemper et al. 2021), biota (Koch et al. 2019; Spaan et al. 2020; Wang et al. 2020), or soils (Wang et al. 2013; Yeung et al. 2013; Tan et al. 2014), yielding a much more comprehensive image. More than a decade later, fluorine sum parameters have been established as a useful supplement to classic target analytical approaches of PFAS (Nakayama et al. 2019) and were implemented for the first time as a sum value “PFAS-total” in the recently revised Drinking Water Directive (2020/2184) by the European Commission.

In contrast, X-ray absorption near-edge structure (XANES) spectroscopy has been widely applied to identify low concentration of element-specific contamination without pre-treatment in environmental samples in the past (Vogel et al. 2016). However, until today, only a small number of fluorine K-edge XANES spectra of metal fluorides were published (Oizumi et al. 1985; Nakai et al. 1986; Hudson et al. 1994; Schroeder and Weiher 2006; Murugesan et al. 2019). The XANES approach enables a penetration depth of approx. 1 µm (at fluorine K-edge energy; depending on the matrix) which is significantly deeper than for X-ray photoelectron spectroscopy (XPS; penetration depth approx. 10 nm) (Tokranov et al. 2019). To the best of our knowledge, only one single fluorine XANES spectrum of perfluorooctanoic acid (PFOA) was published so far (Yan et al. 2021). Thus, the aims of this study were to analyze various PFAS and fluorinated organic compounds by fluorine XANES spectroscopy and to identify PFAS contamination directly in soils and sewage sludges upon application of fluorine XANES spectroscopy without pre-treatment of the samples. Therefore, bulk-XANES spectra of various PFAS, soils, and sewage sludges were collected and analyzed with regard to the findings of the PFAS sum parameter analysis TF/EOF.

## Materials and methods

### Sampling

Four different soil samples from remediation activities in Germany and six additional sewage sludge samples from various wastewater treatment plants in Germany were analyzed. The crude samples were air-dried at room temperature for 30 days and homogenized afterwards. More detailed treatment and investigation data of all soil samples are summarized in the SI (see also Table S1).

### Chemicals and reagents

The following chemicals and materials were used for the experimental work: sodium trifluoromethyl sulfonate (Na-TFMS, 98%, BLD pharma), potassium perfluorobutyl sulfonate (K-PFBS, 98%, Sigma Aldrich), potassium perfluorhexyl sulfonate (K-PFHxS, ≥ 98%, Sigma Aldrich), perfluorooctanesulfonic acid (PFOS, 97%, abcr), sodium trifluoroacetate (Na-TFA, 98%, Alfa Aesar), perfluorooctanoic acid (PFOA, 96%, Sigma Aldrich), perfluorooctanesulfonic acid (PFOS, 97%, abcr), perfluoro-2-propoxypropanoic acid (HFPO-DA, 97%, abcr; carbonic acid of “GenX”), perfluoropentadecane (PFPD, 97%, Fluorochem), 1H,1H,2H,2H-perfluorooctanephosphonic acid (PFOPA, 95%, Sigma Aldrich), 8:2 fluorotelomer alcohol (8:2-FTOH, 97%, Sigma Aldrich), 4-fluorobenzoic acid (4-FBA, 99%, J&K), fluoxetine·HCl (*N*-methyl-*γ*-[4-(trifluoromethyl)phenoxy]benzenepropanamine, > 98%, Fluorochem), tolylfluanid (1,1-dichloro-*N*-[(dimethylamino)sulfonyl]-1-fluoro-*N*-(4-methylphenyl)methanesulfenamide, Pestanal, analytical standard, Sigma Aldrich), perfluoroalkoxy alkanes polymer (PFA, PFA hose, Thermo Scientific), polytetrafluoroethylene (PTFE; Teflon tape Ulith “FRp”), AlF_3_·3H_2_O (≥ 97%, Roth chemicals), CaF_2_ (99.5%, Alfa Aesar), FeF_3_·3H_2_O (Fluorochem), MgF_2_ (99%, 200 mesh, Alfa Aesar), Na_2_SiF_6_ (99%, abcr), GaF_3_·3H_2_O (99.5%, abcr), NaF (p.a., Merck KGaA), KF (> 99%, Roth Chemicals), NH_4_F (p.a., Supelco®, Merck KGaA), and SnF_2_ (99%, Sigma Aldrich). Fluoroapatite (FAp) was precipitated from a NH_4_H_2_PO_4_ (p.a., J.T. Baker) solution with NH_4_F and Ca(NO_3_)_2_∙4H_2_O (both p.a., Sigma Aldrich) and structurally verified by XRD (Gross et al. 2001). For the extraction process, NH_3_ (25%, Suprapur®, Merck KGaA), HCOOH (99–100%, Chemsolute), methanol (MeOH; 99.98%, Rotisolv® HPLC grade, Roth Chemicals), *n*-hexane (Suprasolv, Supelco), acetone (99.5%, p.a., Chemsolute), and WO_3_ (99.9%, Merck KGaA) were used. A Labostar DI 2 system (Siemens Evoqua Water Technologies GmbH) generating ultrapure water (< 0.6 µS/cm) was used for all applications and combustion ion chromatography (CIC) experiments. All utilized SPE cartridges were preassembled Strata PFAS containing a weakly ion exchange (WAX) resin/graphitized carbon black (GCB) combination, purchased by Phenomenex Ltd., Germany.

### Fluorine K-edge XANES spectroscopy

Fluorine K-edge XANES spectra were collected on the PHOENIX II beamline of the Swiss Light Source (SLS, Villigen, Switzerland). All soil and sludge samples were pressed into small pellets for easier sample preparation prior to the measurements, and the experiments were conducted at room temperature under a high vacuum (10^−6^ mbar). The fluorine references were prepared as a thin layer of a few mg spread on a F-free carbon tape. The incoming intensity (*I*_0_) was measured from the total electron yield signal taken from a nickel-coated, 0.5-mm-thick polyester foil. Bulk-XANES spectra were collected from an area of approx. 2 × 3 mm at the sample over the range 660–780 eV in fluorescence mode, using a silicon drift diode (SDD, manufacturer: Ketek). The collected spectra were normalized and background corrected using the Athena software from the Demeter 0.9.26 package (Ravel and Newville 2005). Furthermore, the F K-edge bulk-XANES spectra of the samples were analyzed with linear combination (LC) fitting (Calvin and Furst 2013) of the F reference compounds with the Demeter Athena software. Therefore, processed data of the following F K-edge XANES spectra were used: CaF_2_, MgF_2_, Na_2_SiF_6_, FeF_3_·3H_2_O, AlF_3_·3H_2_O, FAp, NaF, PFOS, Na-TFMS, Na-TFA, fluoxetine, tolylfluanid, and HFPO-DA. The spectral range was set from − 9 to + 15 eV of the fluorine K-edge. The maximum number of compounds in the final LC fit was limited to three, and the sum of the compounds was forced to add up to 100%. From the resulting LC fits, the ones with the lowest goodness of fit *R*-values were chosen.

### Sample extraction and preparation for quantitative analysis

For determination of sum parameter EOF, all samples (soils and sludges) were extracted and prepared according to an optimized method based on previous work by Wilhelm et al. (2019). To assure reproducibility, all samples were prepared in triplicates. Dried solid samples (sludge: 1 g, soil: 2 g) were weighed directly in 50 ml centrifugal polypropylene (PP) tubes, followed by addition of 10 ml of a NH_3_ in MeOH (0.1 M) solution. After that, a standardized extraction process including sonication (15 min), followed by 30 min of vortexing (1500 min^−1^) and subsequent centrifugation (5 min, 4000 min^−1^), was applied to the samples. Following up, the supernatant liquid was carefully decanted to a fresh 50 mL PP tube to avoid transfer of solids from the extraction process. The remaining residues subsequently were extracted in a second run using 10 ml of pure MeOH, followed by the aforementioned extraction process. After decanting the eluates, the combined solutions were carefully concentrated (~ 2 ml) using a gentle constant flow of N_2_. For SPE preparation, all samples were pH adjusted (pH = 4–5) using 0.5% formic acid, diluted to 15 ml with ultrapure water, and eventually centrifuged.

All SPE cartridges were primed applying first 4 ml basic MeOH (0.3% NH_3_), then 4 ml pure MeOH, followed by two subsequent 4 ml steps of ultrapure water. After that, diluted samples were loaded on the solid phases maintaining a constant dripping rate of approx. 1 drop/s. Two washing steps were applied in the following, using 2 × 10 ml of aqueous ammonia solution (0.01%) and 10 ml of deionized water. Subsequent application of a constant vacuum (~ 40 mbar) for approx. 30 min leads to cautious drying of the loaded SPE cartridges. After that, all cartridges were firstly eluted upon slow addition of 2 × 2 ml pure MeOH, followed by three-time addition of 2 ml basic MeOH (0.3% NH_3_) into 15 ml PP vials. Eventually, all SPE cartridges were eluted with 4 ml hexanes, followed by 4 ml of acetone, and collected in fresh 15 ml PP vials. The separated eluates were slowly evaporated to dryness by N_2_ gas and subsequently reconstituted in 2 mL of fresh MeOH.

### Sum parameter analysis (TF/EOF)

All soil and sewage sludge samples were quantified by CIC similar to previously reported work (Gehrenkemper et al. 2021). For TF analysis, pre-homogenized solid samples (40 mg) were weighed directly into ceramic boats and subsequently analyzed by CIC. Prior to analysis, a four-fold excess of powdered WO_3_, serving as flux for high-melting inorganic fluorides, was added to the sample boats. For extractable organically bound fluorine (EOF) analysis, methanol extracts of all samples (500 µL) were injected on quartz wool–filled ceramic boats before being measured by CIC. All liquid samples were handled using a mechanical pipet (Transferpette, Brand GmbH + CO KG). All TF and EOF samples were measured in triplicates to maintain quality in data consistency. In order to quantify the correct absorption volume, an internal standard of known concentration was added to the absorption solution before each combustion step. Quantification of the samples was enabled using an eleven-point calibration curve from 1 to 20 µg/L F^−^ (*R*^2^ = 0.995) for low fluoride-containing samples and a six-point curve from 10 to 500 µg/L F^−^ (*R*^2^ = 0.999) for higher fluoride value detection. Moreover, a ten-point calibration curve from 1 to 1000 mg/L F^−^ (*R*^2^ = 0.997) was prepared for all TF measurements. More detailed description on CIC data acquisition and quality control, including method detection (LOD) and quantification limit (LOQ), are provided in the SI.

## Results and discussion

### Analysis of inorganic fluorides and organofluorine compounds by fluorine K-edge bulk-XANES spectroscopy

**Fig. 1:**
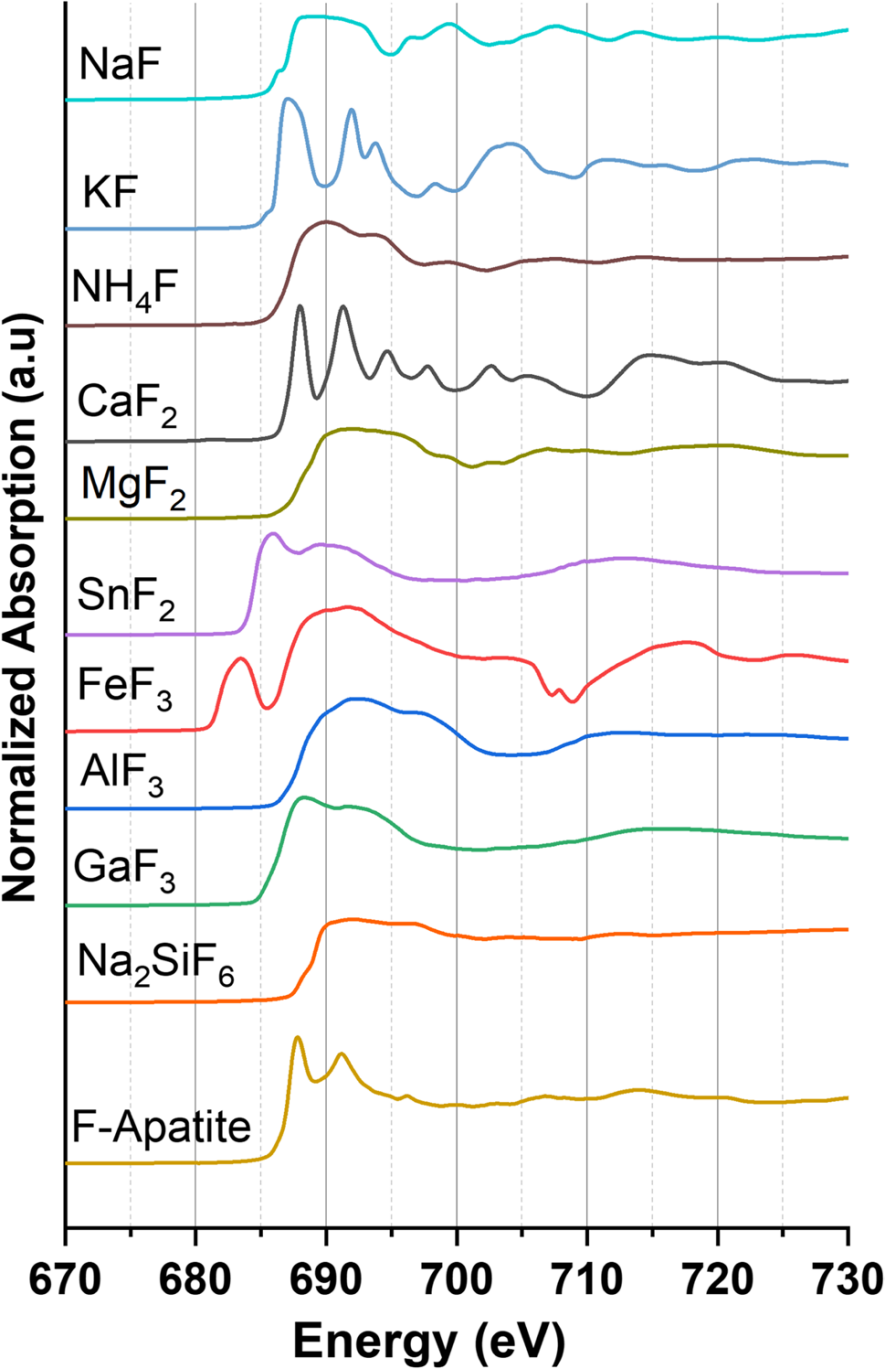
Normalized fluorine K-edge XANES spectra of various inorganic fluorine compounds; fluoroapatite (F-Apatite)

**Fig. 2:**
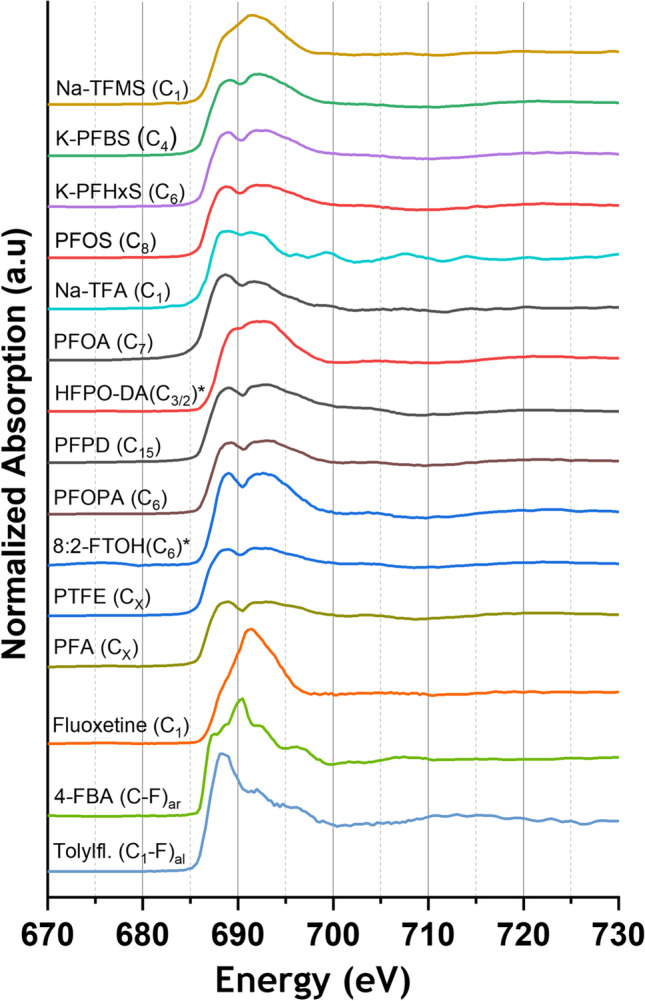
Normalized fluorine K-edge XANES spectra of various fluorinated organic compounds; numbers in brackets show the number of the fully fluorinated carbon atoms per molecule; sodium trifluoromethyl sulfonate (Na-TFMS), potassium perfluorobutyl sulfonate (K-PFBS), potassium perfluorhexyl sulfonate (K-PFHxS), perfluorooctanesulfonic acid (PFOS), sodium trifluoroacetate (Na-TFA), perfluorooctanoic acid (PFOA), perfluoro-2-propoxypropanoic acid (HFPO-DA), perfluoropentadecane (PFPD), 1H,1H,2H,2H-perfluorooctanephosphonic acid (PFOPA), 8:2 fluorotelomer alcohol (8:2-FTOH), polytetrafluorethylene (PTFE), perfluoroalkoxy alkanes polymer (PFA), 4-fluorobenzoic acid (4-FBA)—aromatic C–F bond, tolylfluanid (tolylfl.)—aliphatic C–F bond; * embedded in fluorine-free polyacrylate adhesive

Figures 1 and 2 show the normalized F K-edge XANES spectra of various inorganic and organic fluorine compounds.

While the inorganic fluorine compounds partially have a variety of differently sharp spectral features, the organic fluorine compounds show only two major features. The spectrum of NaF shows a little pre-peak at 686.3, a broad whiteline between 688.0 and 693.0 eV, and several smaller features at 696.3, 699.4, 707.5, and 713.9 eV. For KF, many spectral features at 686.8, 691.7, 693.5, 698.3, and 703.8 eV can be observed. In contrast, the spectrum of NH_4_F shows only little features at 690.0, 694.0, and 699.5 eV. Furthermore, the spectrum of CaF_2_ exhibits a sharp whiteline at 688.0 eV and further features at 691.3, 694.7, 697.8, and 702.6 eV. For fluoroapatite (FAp), the data show a very similar whiteline at 687.8 eV but only two additional spectral features at 691.1 and 696.2 eV. For MgF_2_, a broad whiteline at 691.5 eV and two features at 706.6 and 720.5 eV were recorded, which is in good agreement with previous measurements (Oizumi et al. 1985)_._ In contrast, the spectrum of FeF_3_∙3H_2_O depicts a broad whiteline between 689.0 and 693.0 eV and a large pre-peak at 683.3 eV that was previously detected on other iron fluorides (Murugesan et al. 2019). In addition, interaction with the iron (Fe) L-edge is visible between 705 and 713 eV. The spectrum of AlF_3_∙3H_2_O shows only two major spectral features at 692.0 and 697.5 eV, while Na_2_SiF_6_ has a large whiteline with two maxima at 692 and 696.6 eV and a little feature at 713.3 eV. Eventually, the spectra of GaF_3_ and SnF_2_ display only two broad features at 688.2 and 691.8 and 685.9 and 698.7 eV, respectively.

In summary, almost all recorded PFAS spectra show two very characteristic broad features at 688.5 and 692.0 eV, enabling a clear differentiation from inorganic fluorides (see Figs. 1 and 2). This is in good agreement with the STXM-XANES spectra of PFOA of Yan and coworkers. Moreover, the featured transitions were assigned to F 1 s → σ* C–F transitions of C–F bond in the -CF_2_-CF_2_- chain (Yan et al. 2021). Most notably, this can be regarded in the F K-edge XANES spectrum of the fluoropolymers PTFE and PFA, showing also these specific spectral features. For HFPO-DA, the characteristic broad features are shifted to 689.2 and 692.4 eV. However, due to the high vapor pressure, HFPO-DA and 8:2-FTOH were embedded into polyacrylate adhesive which might explain the shift of the energy maxima. Furthermore, the spectrum of fluoxetine exhibits a very similar pattern compared to the spectrum of Na-TFMS since both compounds contain only one CF_3_ group. In contrast, the mono-fluorinated organic compounds tolylfluanid and 4-FBA show very different shapes of fluorine signals in their F K-edge XANES spectra with a maximum at 688.1 eV (C–F_al_) and 690.3 eV (C–F_ar_), respectively.

In general, the spectral feature at 692.0 eV has a higher intensity for (ultra-)short-chain perfluorosulfonic acids compared with long-chain perfluorosulfonic acids. Therefore, the spectral intensity ratio 688.5 eV/692.0 eV is inversely proportional to the decreasing chain length of the perfluorosulfonic acid group. This might hold true for perfluorocarboxylic acids, since the ultrashort-chain TFA has a higher intensity at 692 eV than the long-chain PFOA (see Fig. 2). Thus, the F K-edge XANES technique allows to differentiate between short- and long-chain PFAS within a given PFAS compound class.

### Investigation of PFAS-contaminated solid matrices by fluorine K-edge XANES spectroscopy

**Fig. 3:**
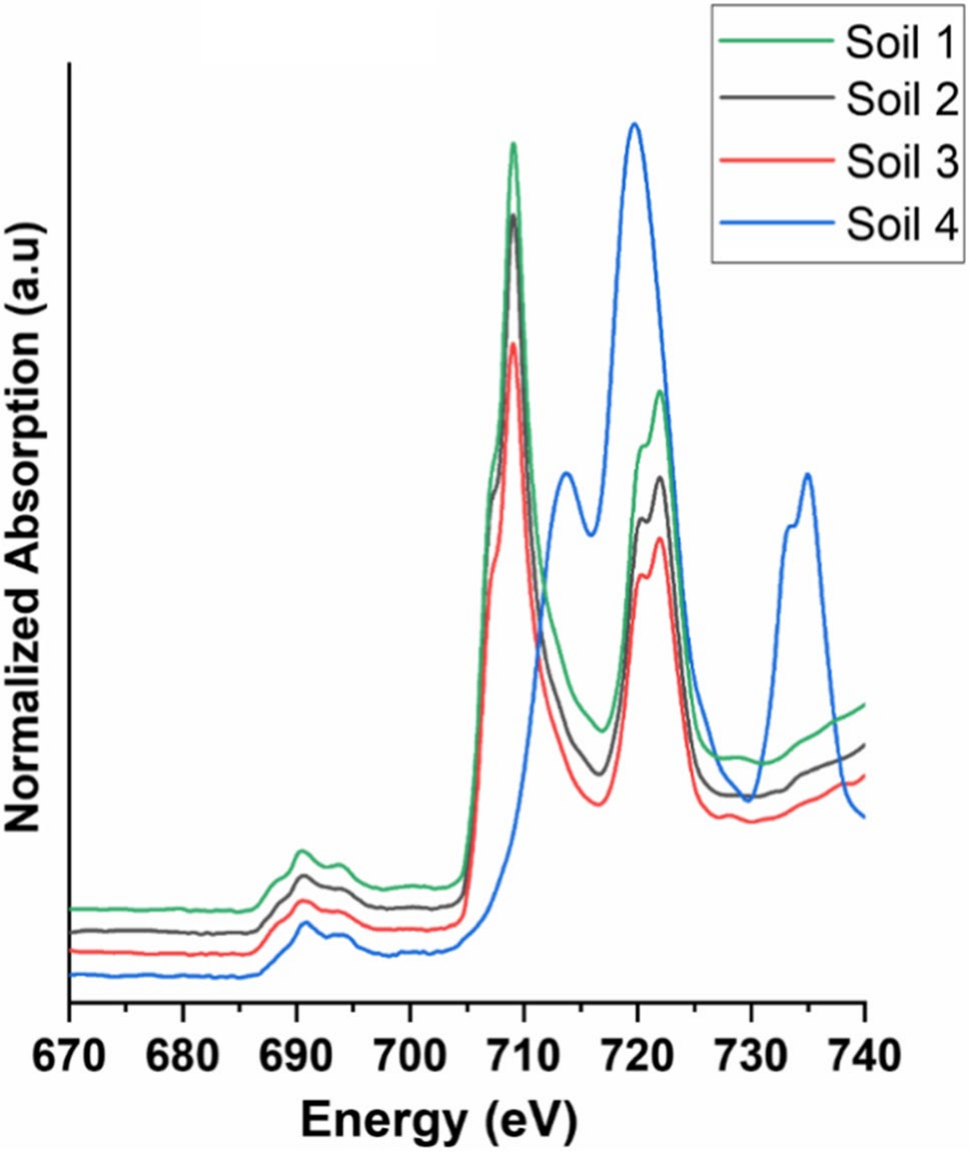
Normalized fluorine K-edge and iron L-edge XANES spectra of the four investigated soil samples

Figure 3 shows the full F K-edge XANES spectra of the analyzed soils (**1–4**), displaying strong features between 705 to 740 eV that can be attributed to absorption bands of the Fe L-edge.

**Fig. 4:**
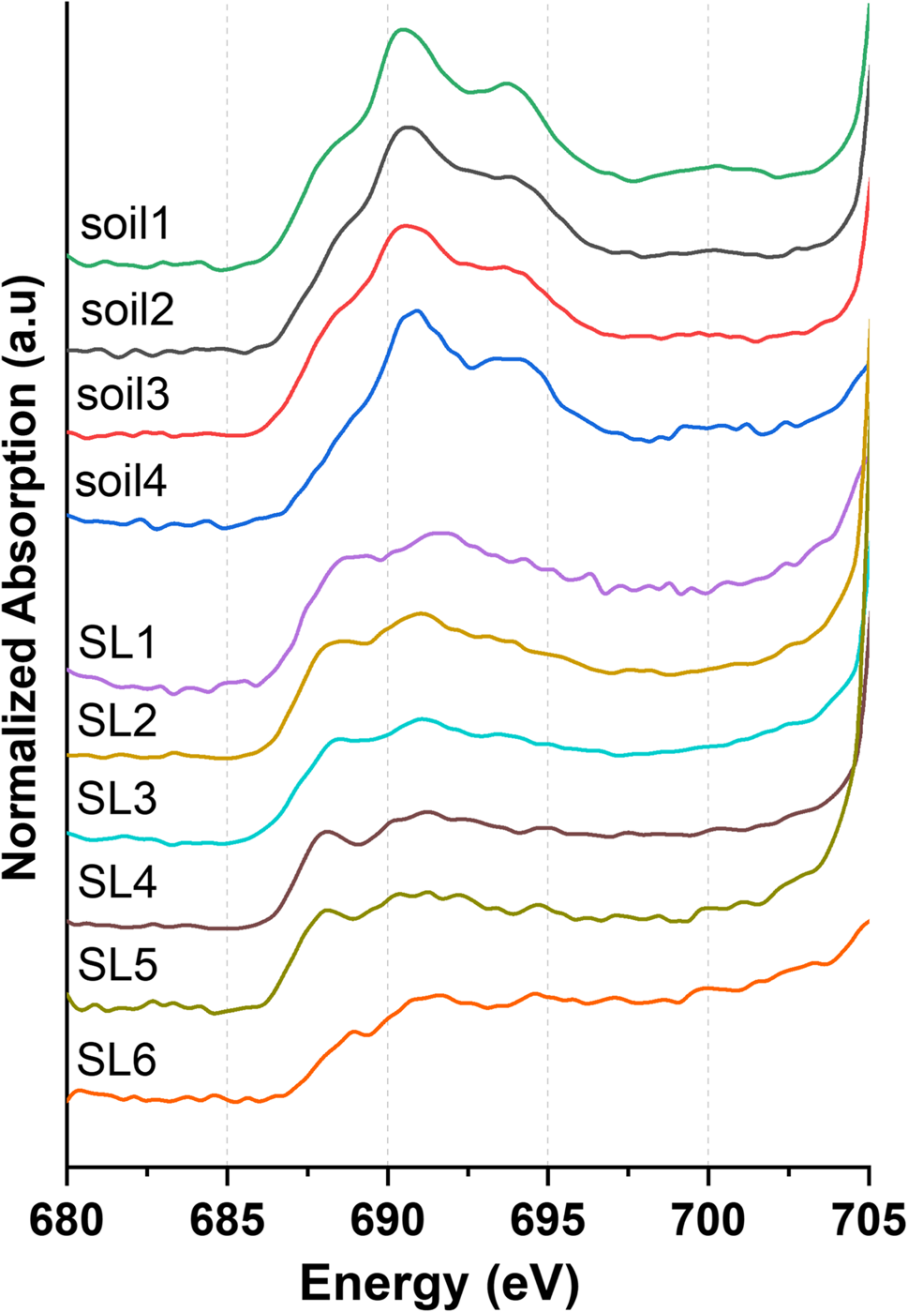
Normalized fluorine K-edge bulk-XANES spectra of the investigated soils (**soil1–4**) and sewage sludges (**SL1–6**)

Usually, the intensity of an element-specific L-edge spectrum is much lower than for the K-edge, but soils naturally contain much more iron than fluorine compounds. Consequently, the higher intensities of Fe L-edge features lead to an overlap in the post-edge region of the F K-edge XANES spectra. Therefore, we focused on the range from 680 to 705 eV in the F K-edge XANES spectra of the analyzed soils and sewage sludges (see Fig. 4).

All analyzed soil samples (Fig. 4 top) show three characteristic features in the F K-edge bulk-XANES spectrum at 688.0, 690.5, and 694.0 eV in different intensities. Besides small alternations between the samples, no characteristic differences could be identified in the respective XANES spectra. In contrast, the sewage sludge samples (Fig. 4 bottom) show completely different XANES spectra with two major features at 688.0 and 691.0 eV. However, the spectral features of the soils and sewage sludges could not be assigned to a specific fluorine compound.

To determine the “detection limit” of this method, we used an approach to analyze quartz spiked with PFOS of various concentrations (1000, 100, 10, 1, and 0.1 mg/kg F; see Fig. S1). With the collection of one single F K-edge XANES spectrum, 1000 and 100 mg/kg PFOS-fluorine in quartz showed a nice fluorine K-edge XANES spectrum. At concentration level 10 mg/kg, the fluorine signal still shows the shape of the PFOS spectrum, but 1 and 0.1 mg/kg PFOS-F could not be detected anymore. Please note that the quality of spectra with a high signal-to-noise ratio can be significantly improved upon addition of several scans of the same sample. Nevertheless, it must be kept in mind that choosing higher photon flux at the beamline and merging of many scans can also decrease the “detection limit” for XANES spectroscopy (Proux et al. 2017). Therefore, it is not straightforward to specify a general detection limit for this method.

### Linear combination analysis of PFAS in fluorine K-edge XANES spectra

In order to analyze the fluorine composition of the samples in more detail, it is possible to perform linear combination (LC) fits of the bulk-XANES spectra based on the recorded spectral information of various inorganic and organic fluorine compounds (Calvin and Furst 2013). This software-based method assembles a library of standards and allows to sum them in various linear combinations, in order to approximate the experimental XANES spectral data as close as possible. LC fitting therefore is very effective to identify the relative number of known constituents that are present in the sample. The results of the performed LC fits are shown in Table 1. All LC fits are classified with the help of the goodness of fit *R*-factor. A lower *R*-factor indicates a better LC fit, whereas an *R*-factor > 0.1 presumes that the model is fundamentally incorrect (Calvin and Furst 2013). *R*-values < 0.02 can be interpreted as “good enough,” while values between 0.02 and 0.10 indicate some major incorrection of the model.

**Table 1:** Summary of the best fits for the linear combination (LC) fittings of the bulk-XANES spectra and the corresponding goodness of fit factors (*R*-factor and reduced *χ*^2^). A lower *R*-factor and reduced *χ*^2^ indicate a better fit

Sample	Best LCF fit	*R*-factor	Reduced *χ*^2^
Soil1	98% fluoxetine + 2% Na_2_SiF_6_	0.042	0.036
	71% MgF_2_ + 13% NaF + 16% CaF_2_	0.060	0.086
Soil2	84% fluoxetine + 13% Na_2_SiF_6_ + 3% MgF_2_	0.033	0.026
	65% MgF_2_ + 20% Na_2_SiF_6_ + 15% FAp	0.084	0.067
Soil3	80% fluoxetine + 20% Na_2_SiF_6_	0.029	0.020
	56% MgF_2_ + 28% FAp + 16% Na_2_SiF_6_	0.072	0.049
Soil4	69% fluoxetine + 31% Na_2_SiF_6_	0.172	0.108
	51% MgF_2_ + 38% Na_2_SiF_6_ + 11% CaF_2_	0.218	0.139
SL1	48% Na-TFMS + 28% FAp + 24% Na_2_SiF_6_	0.054	0.061
	50% FAp + 40% Na_2_SiF_6_ + 10% MgF_2_	0.064	0.049
SL2	47% Na-TFMS + 33% FAp + 20% Na_2_SiF_6_	0.018	0.006
	54% FAp + 35% Na_2_SiF_6_ + 11% MgF_2_	0.026	0.010
SL3	49% Na_2_SiF_6_ + 27% FAp + 24% NaF	0.029	0.009
	44% NaF + 35% Na_2_SiF_6_ + 21% Na-TFMS	0.031	0.010
SL4	48% Na_2_SiF_6_ + 34% NaF + 18% CaF_2_	0.040	0.012
	89% Na_2_SiF_6_ + 11% tolylfluanid	0.104	0.031
SL5	42% Na_2_SiF_6_ + 40% NaF + 18% CaF_2_	0.040	0.012
	42% NaF + 39% Na_2_SiF_6_ + 19% PFOS	0.048	0.015
SL6	92% Na_2_SiF_6_ + 8% NaF	0.144	0.044
	99% Na_2_SiF_6_ + 1% PFOS	0.147	0.046

**Fig. 5:**
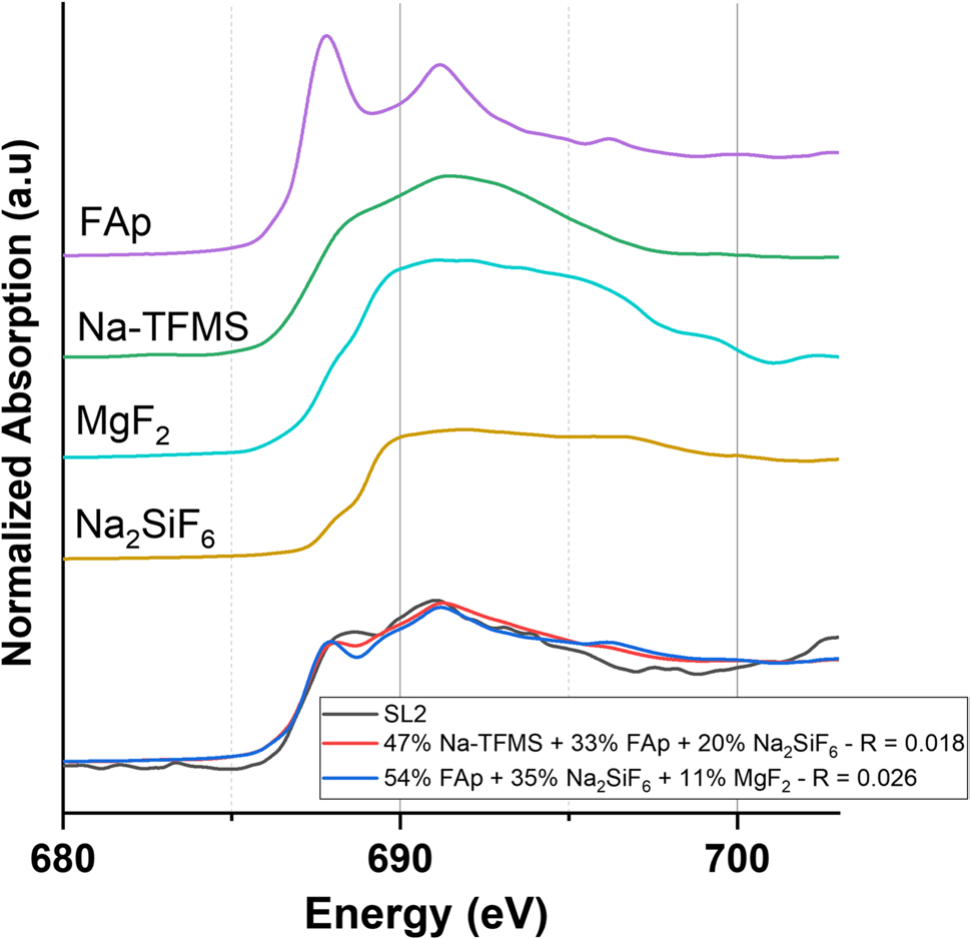
Normalized fluorine K-edge XANES spectrum of **SL2** and the best LC fits (bottom) with the applied reference fluorine compounds (top); fluoroapatite (FAp) and sodium trifluoromethyl sulfonate (Na-TFMS)

For all soils as well as **SL1** and **SL2**, the best LCF fit (Table 1) indicates a PFAS as major F compounds in the sample. Additionally, the fits indicate also minerals such as fluoroapatite (Ca_5_(PO_4_)_3_F) and fluorite (CaF_2_), which belong to the major F-containing minerals in soils (Fuge and Andrews 1988). Due to the existence of only few inorganic fluorides that can occur in natural soils and sludges, the accuracy of the fits is expected to be higher, compared to those for organic PFAS. Some XANES spectra of structurally similar organofluorine compounds are similar, such as for PFOS and PFOPA. Thus, the LC fitting approach is limited in distinguishing between similar organic fluorinated compounds but shows a higher precision with inorganic fluorides. Moreover, some samples exhibit unrealistically large amounts of PFAS in the LC fits in relation to the total fluorine amount, such as for **soil1**. One representative sample for a high PFAS content in the LC fit is **SL2**, displayed in Fig. 5.

Both calculated LC fits of **SL2** are in good approximation to the original spectrum, assigned by the low *R*-value (Calvin and Furst 2013). One fit indicates a 47% Na-TFMS, 33% fluoroapatite, and 20% Na_2_SiF_6_ composition, whereas the other suggests a 54% fluoroapatite, 20% Na_2_SiF_6_, and 11% MgF_2_ distribution. While the first approximation included at least one fluorinated organic compound, resulting in an *R*-value of 0.018, the second fit was based exclusively on inorganic fluorides, yielding a slightly higher *R*-value (0.026). In order to approach the results of the most appropriate fits, we analyze the total amount of organic fluorine (PFAS) in relation to the total fluorine (TF) amount. Consequently, we used combustion ion chromatography (CIC) to determine the TF and the extractable organic fluorine (EOF) content of the investigated samples.

### Total fluorine (TF) and extractable organic fluorine (EOF) analysis of PFAS-contaminated soils and sludges

TF analysis of all samples was conducted by CIC. In order to improve the accuracy of the sum parameter TF, WO_3_ was mixed as fluxing agent to the samples prior to investigation (Shimizu et al. 2015). We found this additive to yield comparable values to the significantly more toxic V_2_O_5_ highlighted in earlier studies on fluorine analysis of environmental samples (Wang et al. 2010). TF analysis was conducted for both, sewage sludge and soil samples, as displayed in Fig. 5 and Table S8.

The TF values of the sludge samples range between 23 and 513 mg/kg and for soils 156 and 1025 mg/kg, most of which can be explained by the presence of inorganic fluorides and non-extractable organic fluorides. The occurrence of inorganic fluorides was also identified with the help of LC fitting of the recorded F XANES spectra (see Fig. 4). Fluoride levels for both matrices are in good agreement with literature data for comparable solid samples (Schuppli 1985; Codling et al. 2014; Geretharan et al. 2018).

**Fig. 6:**
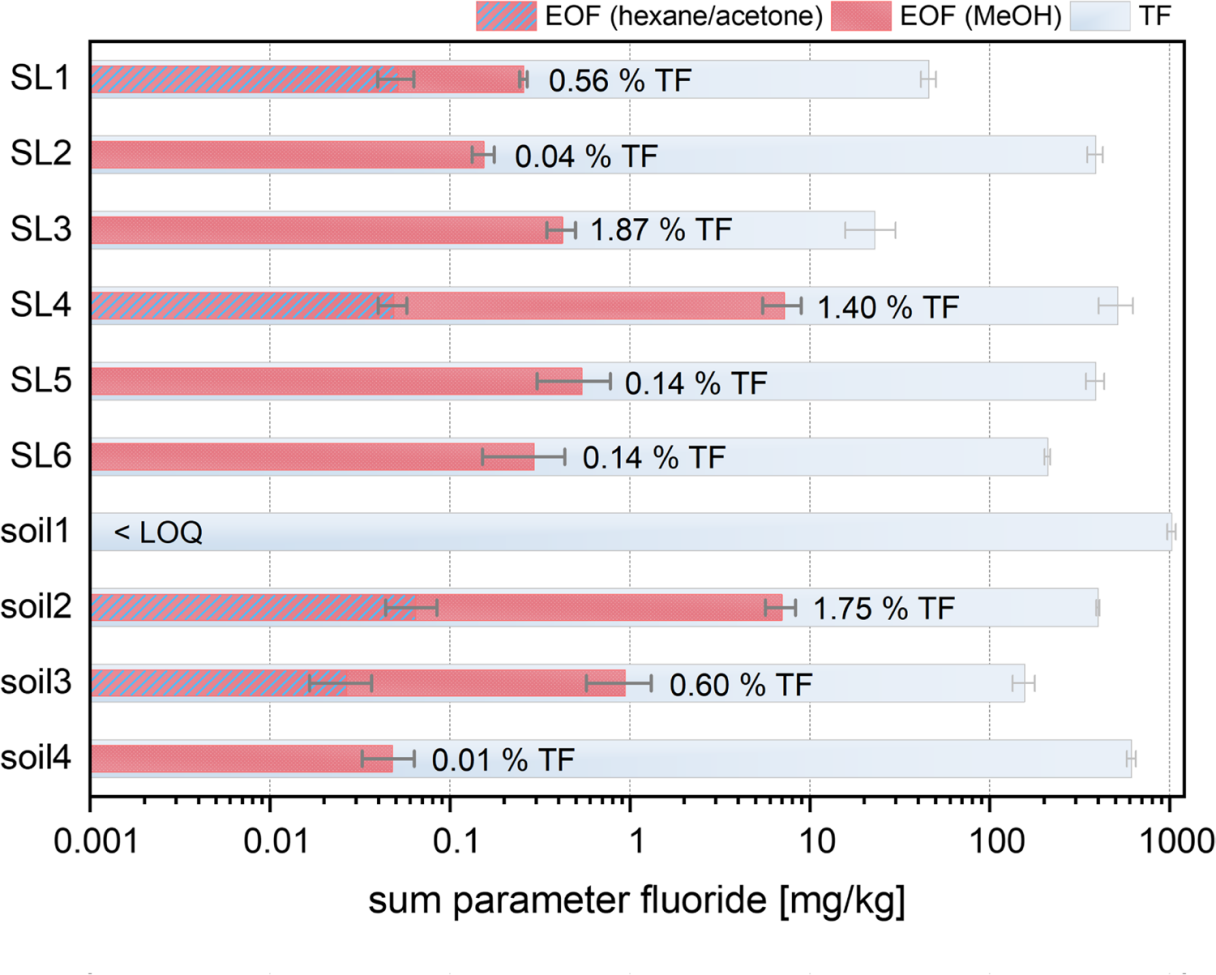
Cumulated EOF values (combined hexanes/acetone and MeOH extraction) over TF values (blue) and respective EOF/TF percentages of the investigated sewage sludge and soil samples (note the log scale). The detected EOF value of **soil1** was below the LOQ. All error bars correspond to the respective standard deviations (*n* = 3)

Whereas several studies considering PFAS sum parameter analysis of contaminated soils were performed during the last decade (Wang et al. 2013; Codling et al. 2014; Tan et al. 2014; Lange et al. 2017), less attention was paid to PFAS sum parameter analysis of polluted sewage sludges (Yeung and Eriksson 2017; Aro et al. 2021). To determine the sum parameter EOF, all soil and sewage sludge samples (**soil1–4**, **SL1–6**) were extracted according to the modified process. In addition to the standard methanol elution step for the WAX cartridge, a second elution mainly for of the GCB phase was applied, using a combination of hexane and acetone. Utilizing various solvents for the SPE cartridges resulted in an unequal elution of the fluoro-organic compounds. The majority of the PFAS compounds showed a higher solubility and mobility in the significantly more polar MeOH, compared to the significantly less polar hexane/acetone eluent (more details in table S9). Only samples **SL1**, **SL4**, as well as **soil2** and **soil3** yielded detectable fluoride values in their respective hexane/acetone fraction. This might be explained by the predominant presence of polar species in the total sample extracts. Figure 6 shows the cumulated EOF values for all studied samples in comparison with their respective TF values.

Comparatively high EOF values were detected for all sewage sludges samples, ranging from 155 µg/kg (**SL2**) up to 539 µg/kg (**SL5**) dry weight (dw). These values are in good conformance with EOF data reported by a recently conducted interlaboratory investigation of EOF sum parameter on sludge samples (Kärrman et al. 2021). Particularly high EOF values were detected for **SL4**, resulting in 7.21 mg/kg dw. Measured EOF values were calculated between 0.04 and 1.84% of the total fluoride amount. Overall, the detected EOF levels clearly exceed the sum of values detected by target analytical methods in previous studies on different sewage sludge samples (Yoo et al. 2009; Gómez-Canela et al. 2012; Gallen et al. 2016; Eriksson et al. 2017; Bolan et al. 2021).

In comparison to sludge samples, soil samples **soil2–4** originate from known PFAS-contaminated sites. Hence, EOF values for **soil2–4** exhibit expectedly high concentrations of 48, 941, and 6985 µg/kg dw, respectively. Their respective EOF/TF percentages vary between 0.01 and 1.75% and are in good agreement with other reported EOF/TF mass balances of contaminated soil (Tan et al. 2014) or water samples (Gehrenkemper et al. 2021; Koch et al. 2021). In contrast, **soil1** is a known PFAS-free soil, yielding an EOF value below the LOQ.

## Conclusion

In summary, we could show that fluorine K-edge XANES spectroscopy can be utilized to detect PFAS in high sample concentrations. The peak intensity ratio 688.5 eV/692.0 eV in the PFAS XANES spectrum can be inversely correlated to the chain length of the perfluorosulfonic acid group and might be also applicable for perfluorocarboxylic acids. However, in environmental soil and sewage sludge samples, the presence of highly concentrated inorganic fluorides significantly decreases the spectral resolution. Linear combination fitting was applied to predict fluorine species in the samples but could not be utilized for identification of specific PFAS. Nonetheless, fluorine K-edge XANES spectroscopy might be considered as an investigative tool in the qualitative analysis of high PFAS concentrations in environmental samples or can be utilized in the analysis of non-inorganic fluoride-containing materials. Compared to other surface detection methods like XPS, fluorine K-edge XANES spectroscopy allows for analysis of much lower concentrated samples, differentiation of inorganic and organic compounds, as well as greater matrix variety, such as soils, sludges, or PFAS-containing consumer products. Moreover, the technique might be suitable for in situ analysis of PFAS degradative processes, like chemically or physically induced PFAS degradation to short-chain compounds or evaluation of PFAS mineralization products. Additionally, the spectroscopic studies were complemented by quantitative sum parameter analysis, yielding a comprehensive picture of the PFAS contamination per sample. Since the detected EOF values were significantly higher than reported in previous target analytical–based studies, the contribution of sum parameter analysis can be considered beneficial.

## Supplementary Information

Below is the link to the electronic supplementary material.

Supplementary file1 (DOCX 164 KB)

## Data Availability

The datasets used and/or analyzed during the current study are available from the corresponding author on reasonable request.
