# Supplementary material for: Investigation of per- and polyfluoroalkyl substances (PFAS) in soils and sewage sludges by fluorine K-edge XANES spectroscopy and combustion ion chromatography
Source: Environ Sci Pollut Res Int. 2021 Dec 3;29(18):26889–99. doi: 10.1007/s11356-021-17838-z (PMC8989862; doi:10.1007/s11356-021-17838-z)
Supplement: Supplementary file 1 — Supplementary file1 (DOCX 164 KB) [file 11356_2021_17838_MOESM1_ESM.docx]

**Supporting Information:**

**Investigation of Per- and Polyfluoroalkyl Substances (PFAS) in Soils and Sewage Sludges by Fluorine K-edge XANES Spectroscopy and Combustion Ion Chromatography**

Philipp Roesch^1^*, Christian Vogel^1^*, Thomas Huthwelker^2^, Philipp Wittwer^1^, Franz-Georg Simon^1^

^1^Bundesanstalt für Materialforschung und -prüfung (BAM), Division 4.3 Contaminant Transport and Environmental Technologies, Unter den Eichen 87, 12205 Berlin, Germany

^2^Paul Scherrer Institute, Swiss Light Sources, 5232 Villigen PSI, Switzerland

**Total SI-Figures: 1**

**Total SI-Tables: 9**

**Total SI-Pages: 12**

**Additional Materials and Methods:**

**Sample preparation**

Soil**1** is uncontaminated topsoil provided by a municipal landfill site in Baden-Württemberg, Germany. Soils **2** and **3** were collected from rural areas around Rastatt in Baden-Württemberg, Germany, where PFAS contamination has been well documented in the last decade(Kotthoff et al. 2020). Soil**4** is a fine fraction collected from a soil washing plant after operating on PFAS contaminated grounds and was kindly made available after several soil remediation treatments. All soils were air-dried for at least 30 days at room temperature yielding a residual moisture content ≤ 2 %. All samples were subsequently homogenized utilizing a jawcrusher and sieves (2 mm). In order to assure consistent distribution, all soils were divided, separately homogenized and filled into small sealable jars.

**Table S1.** Summary of investigated soil samples and their listed properties.

| **number** | **soil** | **Soil1** | **Soil2** | **Soil3** | **Soil4** |
| --- | --- | --- | --- | --- | --- |
| **type of soil** |  | topsoil | PFAS cont. | PFAS cont. | PFAS cont. filter cake |
| **pH value (H_2_O)**  **DIN ISO 10390** |  | 7.67 | 8.47 | 7.12 | 7.72 |
| **original moisture content**  **DIN ISO 12880 [w%]** |  | 19.9 | 4.17 | 16.3 | 27.1 |
| **residual moisture content**  **DIN ISO 12880 [w%]** |  | 2.07 | 3.28 | 1.13 | 1.52 |
| **grain density**  **DIN 66137-2 [g/cm3]** |  | 2.6405 | 2.3778 | 2.5434 | 2.6577 |
| **grain size distribution [%]** | 10-6,3 mm | 14.5 | 0.0 | 13.9 | 0.0 |
|  | 6,3-2 mm | 7.1 | 0.16 | 4.0 | 0.0 |
|  | 2 - 0,63 mm | 4.3 | 22.26 | 10.3 | 0.0 |
|  | 0,63 - 0,2 mm | 5.0 | 63.08 | 43.6 | 0.0 |
|  | 0,2 -0,063 mm | 10.1 | 11.24 | 11.0 | 18.4 |
|  | < 0,063 mm | 58.9 | 3.26 | 17.1 | 81.5 |
| **conductivity [µS/cm]** |  | 180.00 | 100.67 | 150.00 | 225.00 |
| **water absorption**  **DIN 18132 [%]** |  | 53.8 | - | 7.0 | 60.9 |
| **carbonate**  **DIN ISO 10693 [w%]** |  | 7.35 | 0.3 | 1.0 | 7.5 |
| **loss on ignition  DIN ISO 18128 [w%]** |  | 3.03 | 11.43 | 4.68 | 5.08 |
| **methylene blue uptake [mg/g]** |  | 43 | - | 7 | 39 |

**F-XANES detection limit approach**

In order to analyze the “detection level” of fluorine K-edge XANES spectroscopy we spiked fluorine-free quartz (silicon dioxide, washed and calcinated, analytical reagent, Sigma-Aldrich) with various amounts of PFOS: 1000, 100, 10, 1, and 0.1 mg/kg F. Afterwards, one single fluorine K-edge XANES spectrum of each mixture was collected (Fig. S1). According to the spectra shown in figure S1, F bulk-XANES can be used to identify PFOS in samples down to the 10 ppm concentration level. However, it is important to mention that this is not a calibration approach of the method and should only give evidence of the limiting concentration range for a respective sample when the spectral method is applied.





Figure S1: Normalized fluorine K-edge XANES spectra of PFOS spiked quartz samples (1000, 100, 10, 1, 0.1 mg/kg)

**Combustion Ion Chromatography (CIC)**

The CIC combines a combustion and absorption unit (AQF-2100H, GA-210, Mitsubishi Chemical Analytech, Tokyo, Japan) connected to an ion chromatography (IC; ICS Integrion, Thermo Fisher Scientific GmbH, Dreieich, Germany) controlled by the software Chromeleon 7.2.10 (Thermo Fisher Scientific GmbH, Dreieich, Germany). The combustion unit consisted of an autosampler (ASC-210) connected to the induction furnace (AQF-2100H) operating between 1000 and 1050 °C. Prior to combustion, all ceramic boats were prebaked for at least 5 min at 1000 °C to avoid organic contamination. All samples were hydro-pyrolyzed in the horizontal combustion furnace operating at 1050 °C under a flow of O_2_ (300 mL/min), Ar (150 mL/min) using sample specific boat programs (see below). Combustion gases were absorbed in a freshly prepared 3 mM NH_3_ absorption solution, enriched with an internal standard for monitoring the exact absorption volume by ion chromatography. For TF measurements, the water supply level was set to “4” and the medium absorption volume of the GA210 (~16 mL) was selected. For all EOF measurements, the water supply level was set to “2”, combined with the small absorption volume (~11 mL). A 100 µL aliquot (5 µL for TF) was injected into the ion chromatography using Dionex IonPac AG20 (2x50mm) as guard column and Dionex IonPac AS20 (2x250mm) as analytic column, both maintained at a constant column temperature of 30 °C. Chromatographic separation was directed by an automated KOH eluent generator, controlled by an optimized gradient program (5 mM to 50 mM) at a constant flow rate of 0.25 mL/min (see below). Fluoride ions were sensed by a conductivity detector using 50 mM H_2_SO_4_ as suppressor regenerant. For calculation of detected peak areas and fluoride concentrations chromatography data system Chromeleon 7.2.10 (Thermo Fisher Scientific) was used. After combustion of the samples and subsequent quantification of the fluoride amount by IC, the collected raw data were transferred to an external computer for more detailed evaluation in Origin 2020 (OriginLab Corporation).

**Table S2.** Combustion parameters

| **Combustion component** |  |
| --- | --- |
| Combustion device | AQF-2100H, A1 Enviroscience, Mitsubishi Chemical Analytech Co., Ltd. |
| Operating temperature | 1050 °C |
| Ar carrier gas flow | 150 mL/min |
| Ar flow of water supply | 100 mL/min |
| O_2_ flow | 300 ml/min |
| Absorption solution/internal standard | 3.0 mM NH_3_ solution + 2.2 mg/L MeSO_3_H |
| Starting Absorption volume | 8.5 ml (TF); 5.0 mL (EOF) |
| Final Absorption volume | 16 (TF); 11 (EOF) |
| Sample amount | 40 mg (TF); 500 µL (EOF) |
| Water supply level | 4 (TF), 2 (EOF) |

**Table S3.** Ion chromatography parameters

| **Ion chromatography component** |  |
| --- | --- |
| IC-device | ICS Integrion, Thermo Fisher Scientific |
| Detector | conductivity detector |
| Guard column | AG20 2x50mm guard column |
| Analytical column | Dionex IonPac AS20 2x250mm |
| Eluent | gradient KOH |
| Flow rate | 0.25 mL/min |
| Run time | 22 min |
| Column temperature | 30 °C |
| Injection volume | 5 µl (TF), 100 µL (EOF) |
| Suppressor regenerant | 50 mM H_2_SO_4_ |

**Table S4.** EOF boat program, speed level at constant 2 mm/s

| **Pos** | **Time [s]** | **Pos** | **Time [s]** | **Pos** | **Time [s]** | **Pos** | **Time [s]** | **End Time [s]** | **Cool Time [s]** | **Home Time [s]** | **Ar Time [s]** | **O_2_ Time [s]** |
| --- | --- | --- | --- | --- | --- | --- | --- | --- | --- | --- | --- | --- |
| 65 | 30 | 100 | 60 | 130 | 60 | 150 | 60 | 460 | 60 | 120 | 10 | 600 |

**Table S5.** TF boat program

| **Pos** | **Time [s]** | **End Time [s]** | **Cool Time [s]** | **Home Time [s]** | **Ar Time [s]** | **O_2_ Time [s]** |
| --- | --- | --- | --- | --- | --- | --- |
| 90 | 60 | 800 | 60 | 200 | 0 | 800 |

**Table S6.** IC eluent gradient program

| time [min] | eluent concentration [mM] |
| --- | --- |
| 0 | Start, 1.0 |
| 0.1 | 1.0 |
| 0.2 | 2.0 |
| 1.0 | 2.0 |
| 10.0 | 5.0 |
| 10.5 | 5.0 |
| 11.0 | 80.0 |
| 14.0 | 80.0 |
| 14.5 | 1.0 |
| 22.0 | stop run |

**Instrumental Analysis and Quality Control**

CIC - TF

For quantification of the TF values, calibration standards were prepared from a freshly made aqueous NH_4_F stock solution and loaded on ceramic boats of the CIC (500 µl). According to our investigation, ammonium fluoride solutions yielded a more consistent fluoride recovery by combustion in the CIC, compared to NaF solutions. After combustion and quantitative analysis, a ten point calibration curve with concentrations of 1, 2, 5, 10, 20, 50, 100, 200, 500 and 1000 mg/L (R^2^ = 0.9991) was obtained. All TF values were blank corrected by subtraction of the average fluoride amount detected after combustion of WO_3_ (160 mg) loaded sample boats (n=3). In between triplicate analysis, a cleaning step was performed, combusting a blank sample boat under applied measurement conditions. Additionally, quality of the fluoride quantification was guaranteed by determination of the concentration of the internal standard, prior to every sample batch.

CIC - EOF

For quantification of the EOF values, calibration standards were made from a freshly prepared methanolic NH_4_F stock solution and loaded on ceramic boats for combustion in the CIC (500 µl). According to our investigation, ammonium fluoride solutions yielded a more consistent fluoride recovery by combustion in the CIC, compared to NaF solutions. Due to the strong variations in the organo fluorine concentration per sample, two different calibration curves were prepared. In order to ensure maximum precision of all analytical measurements, an eleven point and a six point calibration curve with concentrations of 1, 2, 4, 6, 8, 10, 12, 14, 16, 18, 20 µg/L (R^2^ = 0.995) and 10, 20, 50, 100, 200 and 500 µg/L (R^2^ = 0.999) were recorded, respectively. All EOF values were blank corrected by subtraction of an averaged overall reagent blank value obtained by combustion of a concentrated MeOH solution (analogously to the sample preparation) loaded on sample boats (0.5 mL, n=6). In between triplicate analysis, a cleaning step was performed, combusting a blank sample boat under applied measurement conditions. Reproducibility and reliability of EOF measurements were assured by regularly conducted recovery tests of PFAS stock solutions. Additionally, quality of the fluoride quantification was guaranteed by determination of the concentration of the internal standard, prior to every sample batch.

Limit of detection and quantification LOD/LOQ

Instrumental LOD and LOQ were determined for fluorine analysis using combustion ion chromatography only. Calculation were conducted according to DIN 32645 (German Institute for Standardization 2008). Therefore, ten repeated measurements of ten different blank samples (empty sample boats) were conducted. Subsequently, the standard deviation (SD) was calculated, divided by the slope of the calibration curve (1-20 µg/L F^-^) and multiplied times 3, resulting in the instrumental LOD value. Factor ten was used for the determination of the instrumental LOQ. All measured fluoride values per sample were above LOQ, except for **soil1**.

**Table S7.** Instrumental LOD and LOQ values, determined by CIC (*DIN 32645*)

| Limit of detection [µg/L] fluoride | 1 |
| --- | --- |
| Limit of quantification [µg/L] fluoride | 2 |

**Table S8**. Total fluorine (TF), the summed values of extractable organic fluorine (EOF) of the investigated soils and sludges, their relative standard deviations (RSD) and the percentage of EOF to TF. Standard deviations of added EOF values were calculated via the square root of the added variances of the single values shown in table S9 (sum of variances)

|  | **TF (CIC) [mg/kg]** | **RSD [%]** | **EOF [µg/kg]** | **RSD [%]** | **EOF value/TF value [%]** |
| --- | --- | --- | --- | --- | --- |
| **SL1** | 45.8 ± 4.6 | 10.1 | 256.1 ± 16.9 | 6.6 | 0.56 |
| **SL2** | 386.7 ± 40.6 | 10.5 | 154.3 ± 21.7 | 14.1 | 0.04 |
| **SL3** | 22.9 ± 7.7 | 33.5 | 421.2 ± 76.6 | 18.2 | 1.84 |
| **SL4** | 513.5 ± 118.5 | 23.1 | 7209.4 ± 1748.6 | 24.3 | 1.40 |
| **SL5** | 387.6 ± 7.5 | 1.9 | 539.1 ± 235.2 | 43.6 | 0.14 |
| **SL6** | 209.0 ± 48.8 | 23.4 | 292.5 ± 141.5 | 48.4 | 0.14 |
| **soil1** | 1024.5 ± 59.4 | 5.8 | <LOQ | - | - |
| **soil2** | 398.9 ± 8.9 | 2.2 | 6984.8 ± 1314.0 | 18.8 | 1.75 |
| **soil3** | 155.9 ± 23.7 | 15.2 | 941.0 ± 370.0 | 39.3 | 0.60 |
| **soil4** | 612.2 ± 38.3 | 6.2 | 47.9 ± 15.5 | 32.4 | 0.01 |

**Table S9**. Separated extractable organic fluorine (EOF) values, calculated variances and RSD of the investigated soil and sludge samples, with respect to their extraction medium

|  | **MeOH extraction** | | | **hexane/acetone extraction** | | |
| --- | --- | --- | --- | --- | --- | --- |
|  | **EOF [µg/kg]** | **variance [µg²/kg²]** | **RSD [%]** | **EOF [µg/kg]** | **variance [µg²/kg²]** | **RSD [%]** |
| **SL1** | 204.8 ± 12.1 | 146.5 | 5.9 | 51.3 ± 11.7 | 137.5 | 22.9 |
| **SL2** | 154.3 ± 21.7 | 471.8 | 14.1 | <LOQ | - | - |
| **SL3** | 421.2 ± 76.6 | 5868.2 | 18.2 | <LOQ | - | - |
| **SL4** | 7160.8 ± 1748.5 | 3057368.6 | 24.4 | 48.6 ± 8.8 | 77.1 | 18.1 |
| **SL5** | 539.1 ± 235.2 | 55311.8 | 43.6 | <LOQ | - | - |
| **SL6** | 292.5 ±141.5 | 20028.0 | 48.4 | <LOQ | - | - |
| **soil1** | <LOQ | 0.0 | - | <LOQ | - | - |
| **soil2** | 6920.7 ± 1313.9 | 1726218.8 | 19.0 | 64.1 ± 20.3 | 413.2 | 31.7 |
| **soil3** | 914.5 ± 369.8 | 136777.0 | 40.4 | 26.5 ± 10.0 | 100.1 | 37.7 |
| **soil4** | 47.9 ± 15.5 | 241.1 | 32.4 | <LOQ | - | - |

**References**

German Institute for Standardization (2008). DIN 32645:2008-11 Chemical analysis - Decision limit, detection limit and determination limit under repeatability conditions - Terms, methods, evaluation. Berlin, Beuth. DIN 32645:2008-11: 28.

Kotthoff, M., A. Fliedner, H. Rüdel et al (2020) Per- and polyfluoroalkyl substances in the German environment – Levels and patterns in different matrices. Sci. Total Environ. 740: 140116. doi: 10.1016/j.scitotenv.2020.140116
